# Association between vagus nerve cross-sectional area measured by ultrasound and inflammatory biomarkers in hospitalised patients with COVID-19

**DOI:** 10.5937/jomb0-57970

**Published:** 2026-06-16

**Authors:** Olivera Jovanikić, Goran Stevanović, Ivan Leković, Snježana Vukotić, Nemanja Rančić, Milan Jovanović

**Affiliations:** 1 University of Defence, Faculty of Medicine, Belgrade; 2 Military Medical Academy, Clinic for Neurosurgery, Belgrade; 3 University of Belgrade, Faculty of Medicine, Belgrade; 4 University Clinical Centre of Serbia, Clinic for Infectious and Tropical Diseases, Belgrade; 5 Military Medical Academy, Clinic for Vascular and Endovascular Surgery, Belgrade; 6 Military Medical Academy, Clinic for Urgent Internal Medicine, Belgrade; 7 Center for Clinical Pharmacology, Military Medical Academy, Belgrade; 8 Military Medical Academy, Center for Clinical Pharmacology, Belgrade; 9 Military Medical Academy, Clinic for General and Endocrine Surgery, Belgrade

**Keywords:** COVID-19, vagus nerve, ultrasonography, inflammatory biomarkers, CRP, ferritin, fibrinogen, COVID-19, vagusni nerv, ultrasonografija, inflamatorni biomarkeri, C-reaktivni protein, feritin, fibrinogen

## Abstract

**Background:**

The vagus nerve plays an important role in neuroimmune communication. This study aimed to examine whether systemic inflammatory biomarkers are associated with ultrasound-measured cross-sectional area (CSA) of the vagus nerve in hospitalised patients with COVID-19.

**Methods:**

In this cross-sectional study, 68 hospitalised adults with PCR-confirmed SARS-CoV-2 infection underwent bilateral ultrasound assessment of vagus nerve CSA. For each nerve, CSA was calculated as the mean of three measurements. Serum levels of C-reactive protein (CRP), fibrinogen, and ferritin were recorded. CSA values and inflammatory biomarkers were classified as within or outside predefined reference ranges. Binary logistic regression was used to assess associations between inflammatory biomarkers and vagus nerve CSA classified as outside the reference range. Receiver operating characteristic (ROC) analysis was performed. As a secondary exploratory analysis, a multilayer perceptron neural network was constructed to evaluate whether the same biomarkers could classify vagus nerve CSA as within or outside the reference range.

**Results:**

Mean vagus nerve CSA was 1.32±0.30 mm2 on the right side and 1.20±0.28 mm2 on the left side. Inflammatory biomarkers were significantly associated with right vagus nerve CSA classified as outside the reference range in logistic regression, whereas no significant association was observed for the left vagus nerve. The regression model showed acceptable discriminative performance for the right vagus nerve (AUC=0.76, p=0.018). The exploratory neural network model showed similar classification performance.

**Conclusions:**

Systemic inflammatory biomarkers were associated with right vagus nerve CSA classified as outside the reference range on ultrasound, but not with left vagus nerve CSA, in hospitalised patients with COVID-19. These findings suggest a possible link between systemic inflammation and ultrasound-detectable variation in vagus nerve morphology. Larger studies are needed to confirm these findings and clarify their biological significance.

## Abstract (sr)

**Uvod:**

Vagusni nerv ima važnu ulogu u neuroimunološkoj komunikaciji. Cilj ove studije bio je da se ispita da li su sistemski inflamatorni biomarkeri povezani sa ultrazvučno izmerenom površinom poprečnog preseka (PPP) vagusnog nerva kod hospitalizovanih pacijenata sa COVID-19.

**Metode:**

U ovoj studiji preseka, 68 hospitalizovanih odraslih osoba sa PCR-potvrđenom SARS-CoV-2 infekcijom podvrgnuto je obostranom ultrazvučnom merenju površine poprečnog preseka (CSA) vagusnog nerva. Za svaki nerv, CSA je izračunata kao srednja vrednost tri merenja. Zabeleženi su serumski nivoi C-reaktivnog proteina (CRP), fibrinogena i feritina. Vrednosti CSA i inflamatornih biomarkera klasifikovane su kao unutar ili van unapred definisanih referentnih opsega. Binarna logistička regresija korišćena je za procenu povezanosti između inflamatornih biomarkera i CSA vagusnog nerva klasifikovane kao van referentnog opsega. Izvršena je ROC analiza (Receiver Operating Characteristic). Kao sekundarna eksploratorna analiza, konstruisana je višeslojna perceptronska neuronska mreža radi procene da li isti biomarkeri mogu klasifikovati CSA vagusnog nerva kao unutar ili van referentnog opsega.

**Rezultati:**

Prosečna površina poprečnog preseka (CSA) vagusnog nerva iznosila je 1,32±0,30 mm2 na desnoj strani i 1,20±0,28 mm2 na levoj strani. Inflamatorni biomarkeri bili su značajno povezani sa CSA desnog vagusnog nerva klasifikovanom kao van referentnog opsega u logističkoj regresiji, dok za levi vagusni nerv nije utvrđena značajna povezanost. Regresioni model pokazao je prihvatljive diskriminativne performanse za desni vagusni nerv (AUC=0,76; p=0,018). Model eksploratorne neuronske mreže pokazao je slične klasifikacione performanse.

**Zaključak:**

Inflamatorni biomarkeri bili su povezani sa ultrazvučno izmerenom CSA desnog vagusnog nerva klasifikovanom kao van referentnog opsega kod hospitalizovanih pacijenata sa COVID-19. Ovi nalazi ukazuju na moguću povezanost između sistemske upale i ultrazvučno utvrđenih varijacija u površini poprečnog preseka vagusnog nerva. Potrebne su obimnije studije radi potvrde ovih nalaza i razjašnjenja njihovog biološkog značaja.

## Introduction

The vagus nerve is a major component of neuroimmune communication and plays an important role in regulating inflammatory responses. Through its extensive afferent and efferent connections, the vagus nerve participates in bidirectional signalling between peripheral organs, the immune system, and the central nervous system. This neuroimmune interaction has been described within the framework of the inflammatory reflex, a physiological mechanism that senses peripheral inflammation and modulates cytokine responses [1][2][3][4][5][6]. Experimental and clinical studies have shown that vagal pathways may regulate immune responses through cholinergic anti-inflammatory signalling and modulation of cytokine production [1][2][3][4][5][7]. Although the functional role of the vagus nerve in inflammatory regulation has been studied extensively, little is known about whether systemic inflammation is associated with measurable structural changes in the vagus nerve in acute infectious conditions [1][2][5].

A variable characterises coronavirus disease 2019 (COVID-19), but it often involves a pronounced systemic inflammatory response. Elevated levels of inflammatory biomarkers, including C-reactive protein (CRP), fibrinogen, ferritin, and several cytokines, have been associated with disease severity and adverse clinical outcomes in patients with SARS-CoV-2 infection [8][9][10][11][12][13][14][15]. Among these biomarkers, CRP fibrinogen, and ferritin are widely available in routine clinical practice and are commonly used as indicators of inflammatory burden in hospitalised patients with COVID-19 [8][9][10][11][12][13]. Severe forms of COVID-19 have been associated with dysregulated cytokine responses and excessive inflammatory activation, often referred to as a cytokine storm [16][17][18][19]. However, although these markers reflect systemic inflammation, it remains unclear whether they are associated with ultrasound-detectable changes in peripheral neuroimmune structures, such as the vagus nerve.

High-resolution ultrasound is a non-invasive and clinically accessible method for assessing peripheral nerve morphology. Measurement of nerve cross-sectional area (CSA) is commonly used in neuromuscular ultrasound as a quantitative parameter reflecting nerve size. Standardised sonographic protocols for vagus nerve assessment have been described, and published reference values provide a basis for identifying CSA values within or outside expected ranges [20][21]. Ultrasound assessment of the vagus nerve has also been explored in several neurological conditions, demonstrating that sonographic measurement of vagus nerve CSA may provide useful structural information in clinical research [20][21][22].

The biological rationale for such an association is plausible, although still insufficiently understood. Because the vagus nerve is involved in neuroimmune signalling, systemic inflammation during acute viral infection may be accompanied by measurable variation in vagus nerve CSA. At present, however, evidence directly linking inflammatory biomarkers and ultrasound-assessed vagus nerve CSA in hospitalised patients with COVID-19 remains limited.

Therefore, this study aimed to examine whether systemic inflammatory biomarkers are associated with ultrasound-measured cross-sectional area of the vagus nerve in hospitalised patients with COVID-19. In particular, we evaluated the relationships between CRP fibrinogen, and ferritin and CSA values of the right and left vagus nerves, using logistic regression as the primary analytical approach and an exploratory neural network model as a secondary analysis.

**Figure 1:**
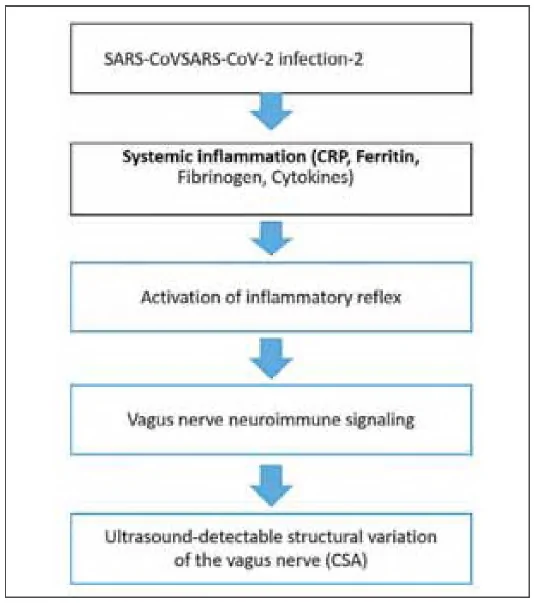
Proposed neuroimmune pathway linking systemic inflammation and vagus nerve structural variation in COVID-19.<br>Systemic inflammation during SARS-CoV-2 infection may activate the inflammatory reflex and vagus nerve neuroimmune signalling, potentially contributing to ultrasound-detectable variation in vagus nerve cross-sectional area.

The proposed neuroimmune pathway linking systemic inflammation and structural variation in the vagus nerve in COVID-19 is illustrated in Figure 1.

## Materials and methods

### Study design and participants

This prospective, cross-sectional observational study included 68 hospitalised adult patients with SARS-CoV-2 infection confirmed by polymerase chain reaction (PCR). The study was conducted between October 2020 and April 2021 after approval by the local institutional ethics committee. Written informed consent was obtained from all participants before inclusion.

Patients were enrolled at the time of hospital admission. Individuals with a history of neurological disease, previous neck surgery or trauma, or other conditions potentially affecting vagus nerve morphology were excluded. Demographic and clinical characteristics, including age, sex, smoking status, comorbidities, and ongoing therapies, were recorded.

### Ultrasound assessment of the vagus nerve

Ultrasound examination of the vagus nerve was performed immediately after admission using a high-resolution ultrasound system (Toshiba Aplio 500, Japan) equipped with a linear probe operating at 12-18 MHz. The examination was performed using a standardised protocol for peripheral nerve imaging [20][23].

Patients were examined in the supine position with mild neck extension. The vagus nerve was identified within the carotid sheath and visualised in both longitudinal and transverse planes before measurement. Measurements were performed at the level of the carotid bifurcation or the distal segment of the common carotid artery [20][21][22].

Care was taken to avoid compression of the vagus nerve by maintaining clear visualisation of the internal jugular vein during the examination. Power Doppler mode was used to exclude adjacent vascular structures when necessary.

The cross-sectional area (CSA) of each vagus nerve was measured by tracing the inner border of the hyperechoic epineural rim. For each nerve, three consecutive CSA measurements were obtained during the same ultrasound examination, and the mean of the three measurements was used for analysis to reduce random measurement variability [20]. CSA values were expressed in square millimetres (mm^2^).

Reference ranges for vagus nerve CSA were based on previously published ultrasound studies in adult populations [20][21][22]. According to these references, CSA values of 1.76-2.70 mm^2^ for the right vagus nerve and 1.44-2.28 mm^2^ for the left vagus nerve were considered within normal limits. CSA values were therefore categorised as within the reference range (0) or outside the reference range (1).

### Inflammatory biomarkers

Blood samples were collected on the same day as the ultrasound examination. Serum levels of C-reactive protein (CRP), fibrinogen, and ferritin were measured using standard hospital laboratory methods [8][9][10][11][12][13].

For analytical purposes, each biomarker was categorised as within the reference range (0) or outside the reference range (1) according to institutional laboratory reference values.

### Statistical analysis

Statistical analyses were performed using SPSS version 26 (IBM Corp., Armonk, NY, USA).

Continuous variables were summarised using mean and standard deviation (SD) for normally distributed data and median with interquartile range for non-normally distributed data. Categorical variables were expressed as frequencies and percentages.

Binary logistic regression was used as the primary analytical method to evaluate associations between inflammatory biomarkers (CRP fibrinogen, and ferritin) and vagus nerve CSA classified as outside the reference range. Model discrimination was assessed using receiver operating characteristic (ROC) analysis and the area under the ROC curve (AUC).

As a secondary exploratory analysis, a multilayer perceptron neural network model was constructed to evaluate whether the same inflammatory biomarkers could classify vagus nerve CSA as within or outside the reference range. The neural network model included CRP, fibrinogen, and ferritin as input variables and vagus nerve CSA classification as the output variable.

A p-value <0.05 was considered statistically significant.

## Results

### Study population

A total of 68 hospitalised patients with PCR-confirmed SARS-CoV-2 infection were included in the study. The mean age of the participants was 53±14 years (range 20-82 years). The study population included 35 women (51.5%) and 33 men (48.5%), with no significant sex difference in distribution. Fourteen patients (20.6%) were smokers. Most patients (95.4%) were receiving acetylsalicylic acid therapy before the ultrasound examination. No patients were receiving medications known to affect CRP, fibrinogen, or ferritin levels significantly.

Baseline demographic and clinical characteristics of the study population are presented in Table 1.

**Table 1:** Baseline characteristics of the study population.

Variable	Value
Number of patients	68
Age, years (mean ± SD)	53±14
Age range, years	20-82
Sex	
Female, n (%)	35 (51.5)
Male, n (%)	33 (48.5)
Smoking status	
Smokers, n (%)	14 (20.6)
Acetylsalicylic acid therapy, n (%)	65 (95.4)
Vagus nerve CSA	
Right vagus nerve CSA (mm^2^), mean ± SD	1.32±0.30
Left vagus nerve CSA (mm^2^), mean ± SD	1.20±0.28
CSA classification (right vagus nerve)	
Within reference range, n (%)	8 (11.8)
Outside reference range, n (%)	60 (88.2)
CSA classification (left vagus nerve)	
Within reference range, n (%)	20 (29.4)
Outside reference range, n (%)	48 (70.6)
Inflammatory biomarkers	
CRP within reference range, n (%)	8 (11.8)
CRP outside reference range, n (%)	60 (88.2)
Fibrinogen within reference range, n (%)	15 (22.1)
Fibrinogen outside reference range, n (%)	53 (77.9)
Ferritin within reference range, n (%)	6 (8.8)
Ferritin outside reference range, n (%)	62 (91.2)

### Vagus nerve cross-sectional area

The mean cross-sectional area (CSA) of the vagus nerve was 1.32±0.30 mm^2^ on the right side and 1.20±0.28 mm^2^ on the left side. According to the selected reference ranges, 11.8% of patients had CSA values within the normal range on the right side. In comparison, 88.2% had CSA values outside the reference range. For the left vagus nerve, 29.4% of patients had CSA values within the reference range, while 70.6% had values outside it.

**Figure 2:**
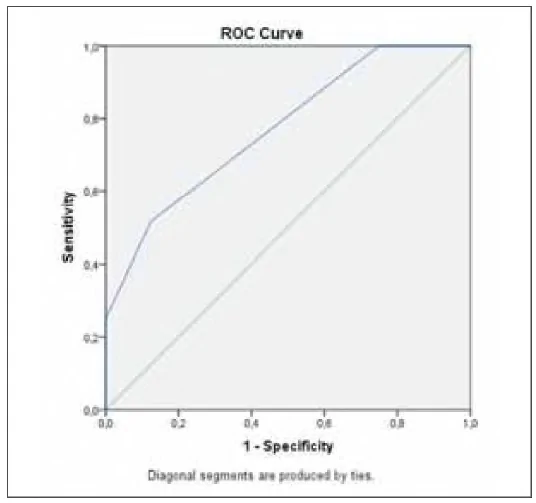


**Figure 3:**
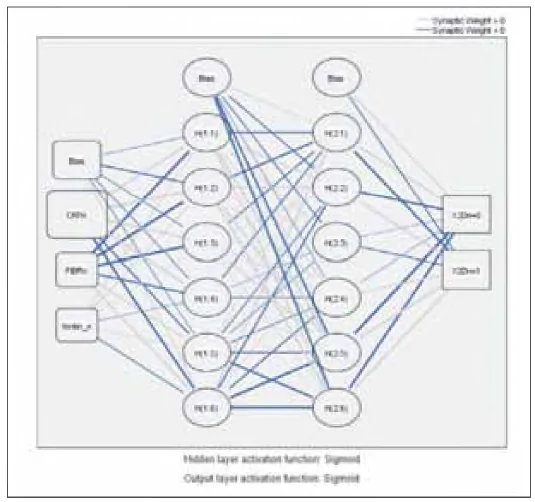


No statistically significant difference was observed between the CSA values of the right and left vagus nerves in the overall population (Mann-Whitney test, p = 0.539). Similarly, CSA values did not differ significantly between male and female participants (right vagus nerve p=0.742; left vagus nerve p=0.632). Figure 2 Figure 3

### Inflammatory biomarkers

Serum inflammatory biomarkers showed that 11.8% of patients had CRP values within the reference range, while 88.2% had elevated values. For fibrinogen, 22.1% of patients had values within the reference range, while 77.9% had values outside it. Ferritin values were within the reference range in 8.8% of patients, while 91.2% were outside it.

### Logistic regression analysis

Binary logistic regression analysis was performed to evaluate the association between inflammatory biomarkers (CRP, fibrinogen, and ferritin) and vagus nerve CSA classified as outside the reference range. The regression model demonstrated a significant association between inflammatory biomarkers and right vagus nerve CSA classified as outside the reference range (Omnibus test c^2 ^= 14.193, p = 0.003) and explained a moderate proportion of variance (Nagelkerke R^2^ = 0.365). The model showed acceptable discriminative performance with an area under the ROC curve (AUC) of 0.76 (p=0.018).

Within the model, CRP showed the strongest contribution, while fibrinogen and ferritin demonstrated weaker relative contributions.

### Exploratory neural network analysis

**Figure 4:**
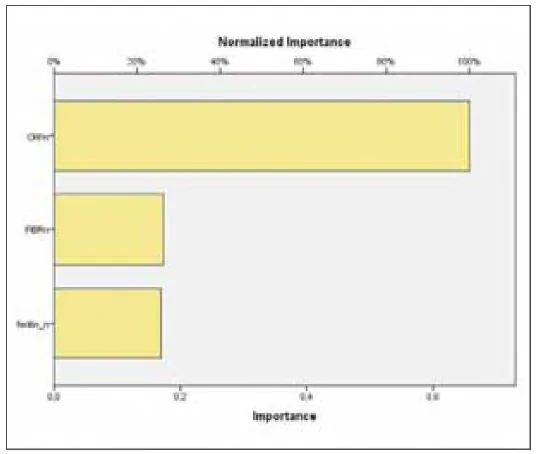
Importance

As a secondary exploratory analysis, a multilayer perceptron neural network model was constructed using CRP fibrinogen, and ferritin as input variables and right vagus nerve CSA classification as the output variable. The dataset was randomly split into training (n=47) and test (n = 21) sets. The model achieved classification accuracies of 91.5% on the training set and 90.5% on the test set. ROC analysis yielded an AUC of 0.76 (p = 0.018), similar to the logistic regression model. Figure 4 Table 2

**Table 2:** ROC analysis of logistic regression and neural network models for right vagus nerve CSA classification.

Model	AUC	p-value
Logistic regression model	0.76	0.018
Neural network model	0.76	0.018

### Left vagus nerve analysis

When the same analytical approaches were applied to the left vagus nerve, neither the logistic regression model nor the neural network model demonstrated statistically significant classification performance.

## Discussion

In this study, we examined the association between systemic inflammatory biomarkers and ultrasound-measured cross-sectional area (CSA) of the vagus nerve in hospitalised patients with COVID-19. The main finding was that inflammatory biomarkers were significantly associated with right vagus nerve CSA classified as outside the reference range, whereas no such association was observed for the left vagus nerve. The regression model demonstrated moderate discriminative performance, and similar classification results were observed in the exploratory neural network analysis.

The vagus nerve plays an important role in neuroimmune communication and in the regulation of inflammatory responses. Previous experimental and clinical studies have described the involvement of the vagus nerve in the so-called inflammatory reflex, a physiological mechanism that modulates cytokine production and immune responses through bidirectional signalling between the peripheral immune system and the central nervous system [1][2][3][5][6]. This reflex is considered one of the key pathways linking the nervous system with immune regulation and systemic inflammatory responses [7][24]. However, most of the existing literature has focused on the functional aspects of vagal signalling rather than the nerve's structural characteristics.

Ultrasound has recently emerged as a noninvasive tool for evaluating peripheral nerve morphology, including the vagus nerve. Several studies have reported reference values for the cross-sectional area of the vagus nerve and have demonstrated variability in CSA depending on factors such as sex, age, and measurement protocol [20][21][22][23]. In many populations, the CSA of the right vagus nerve has been reported to be slightly larger than that of the left nerve [20][21][22]. In the present study, we did not observe a statistically significant difference in CSA between the right and left vagus nerves in the overall population. Nevertheless, only the right vagus nerve CSA showed an association with inflammatory biomarkers.

The reasons for this apparent asymmetry remain uncertain. Anatomical and functional differences between the right and left vagus nerves have been described in previous studies, including differences in their peripheral distribution and central projections [20][21][22]. It is therefore possible that systemic inflammatory processes may affect the two nerves differently, although this hypothesis requires further investigation.

Another notable observation in our cohort was the high proportion of patients with CSA values outside the selected reference ranges. This finding may reflect differences between the hospitalised COVID-19 population and previously studied reference populations [20][21][22][23]. However, the clinical significance of these observations remains uncertain, and caution is required when interpreting CSA values outside published reference intervals. Systemic inflammatory responses and autonomic nervous system involvement have been described in patients with COVID-19. They may contribute to neuroimmune alterations affecting peripheral nerves [4][16][17].

Among the inflammatory biomarkers evaluated in this study, CRP showed the strongest association with vagus nerve CSA, whereas fibrinogen and ferritin contributed less in the regression model. CRP is a widely used marker of systemic inflammation and has been repeatedly associated with disease severity in COVID-19 [8][9][10][11]. Elevated ferritin and fibrinogen levels have also been reported as indicators of excessive inflammatory activation and cytokine dysregulation during SARS-CoV-2 infection [10][11][12][13]. Similarly, increased fibrinogen concentrations reflect inflammatory and coagulation disturbances commonly observed in patients with severe COVID-19 [12][13]. The observed relationship between CRP levels and vagus nerve CSA may therefore reflect the overall inflammatory burden present in these patients rather than a direct causal mechanism.

More broadly, SARS-CoV-2 infection is characterised by activation of multiple inflammatory pathways, including cytokine-mediated immune responses involving IL-6 and other pro-inflammatory mediators [14][15][16][17]. These systemic inflammatory processes may interact with neuroimmune regulatory pathways involving the vagus nerve and the cholinergic anti-inflammatory pathway [18][19].

The exploratory neural network analysis yielded classification performance comparable to that of logistic regression. Because of the relatively small sample size, this analysis should be interpreted cautiously and considered primarily as an exploratory complement to the regression analysis rather than as a definitive predictive model.

Several limitations should be considered when interpreting the results of this study. First, the study included a relatively small number of patients from a single centre. Second, the cross-sectional design does not allow for causal or temporal conclusions regarding the relationship between systemic inflammation and vagus nerve CSA. Third, ultrasound assessment evaluates nerve size but does not directly measure vagal function or detailed nerve microstructure [20][23].

Despite these limitations, the present study suggests that systemic inflammatory biomarkers may be associated with ultrasound-measured vagus nerve CSA in patients with COVID-19. Previous reports have suggested that modulation of vagus nerve activity may influence inflammatory responses and potentially represent a therapeutic target in inflammatory diseases, including COVID-19 [7][24][25]. Future studies with larger populations and longitudinal designs will be necessary to determine whether these observations reflect transient inflammatory changes, patient-specific anatomical variation, or clinically relevant neuroimmune interactions.

## Conclusion

In conclusion, this study suggests an association between systemic inflammatory biomarkers and right vagus nerve CSA classified as outside the reference range on ultrasound in hospitalised patients with COVID-19. These findings suggest that systemic inflammatory status may be reflected in ultrasound-detectable variation of vagus nerve size. Further studies with larger cohorts and longitudinal designs are required to determine the biological and clinical significance of these observations.

## Dodatak

### Conflict of interest statement

All the authors declare that they have no conflict of interest in this work.

### List of abbreviations

CSA, cross-sectional area;<br>CRPC-reactive protein
